# Liquiritin, as a Natural Inhibitor of AKR1C1, Could Interfere With the Progesterone Metabolism

**DOI:** 10.3389/fphys.2019.00833

**Published:** 2019-07-03

**Authors:** Chenming Zeng, Difeng Zhu, Jun You, Xiaowu Dong, Bo Yang, Hong Zhu, Qiaojun He

**Affiliations:** ^1^Hangzhou Institute of Innovative Medicine, College of Pharmaceutical Sciences, Zhejiang University, Hangzhou, China; ^2^College of Pharmaceutical Sciences, Center for Drug Safety Evaluation and Research of Zhejiang University, Zhejiang University, Hangzhou, China; ^3^Zhejiang Cancer Hospital, Hangzhou, China

**Keywords:** liquiritin, AKR1C1, inhibitor, progesterone metabolism, pre-term birth prevention

## Abstract

Low progesterone level is always linked with pre-term birth. Therefore, maintaining of progesterone level is vital during pregnancy. Aldo-keto reductase family one member C1 (AKR1C1) catalyzes the reduction of progesterone to its inactive form of 20-alpha-hydroxy-progesterone and thus limits the biological effect of progesterone. In our effort to identify the natural compound that would specifically inhibit AKR1C1, liquiritin was found to be a selective and potent inhibitor of AKR1C1. Kinetic analyses in the S-(+)-1,2,3,4-tetrahydro-1-naphthol (s-tetralol) catalyzed by AKR1C1 in the presence of the inhibitors suggest that liquiritin is a competitive inhibitor by targeting the residues Ala-27, Val-29, Ala-25, and Asn-56 of AKR1C1. In HEC-1-B cells, treatment with liquiritin results in 85.00% of reduction in progesterone metabolism, which is mediated by AKR1C1 enzymatic activity. Overall, our study not only identify liquiritin as an inhibitor against AKR1C1, but also reveal that liquiritin may be served as a potential intervention strategy for preventing pre-term birth caused by low progesterone level.

## Introduction

Progesterone, a natural female hormone, is essential for pregnancy maintenance (Csapo et al., 1971; Spencer and Bazer, 2002; Romero et al., 2014). The progesterone deficiency in serum levels is closely associated with pre-term birth (Tan et al., 2012). For several decades, extensive efforts has been taken to explore the potential application of progesterone supplementation to prevent pre-term birth (da Fonseca et al., 2003; Norwitz and Caughey, 2011). Progesterone supplements can be taken in various ways, including vaginal gels, vaginal suppositories, vaginal inserts, oral capsules and injections (Cicinelli et al., 2000). Because progesterone affects the body in several different ways, there can be a lot of side risks associated with supplement use, includes elevating breast cancer and heart diseases risks as well as fetal malformations (Rock et al., 1985; Chlebowski et al., 2015).

Aldo-keto reductase family 1 member C1 (AKR1C1) has a major role in progesterone metabolism (Zhang et al., 2000; Rizner et al., 2006; Hevir et al., 2011). AKR1C1 is a member of human hydroxysteroid dehydrogenases which belongs to the family of NADPH-dependent cytosolic oxidoreductases (Penning, 1997). These enzymes share a high percentage of amino-acid sequence identity that ranges from 84.00 to 98.00%. AKR1C1 can convert progesterone to its inactive form of progestin, 20-alpha-hydroxy-progesterone. As metabolite of progesterone has a quite low affinity for the progesterone receptors, AKR1C1 therefore plays a critical role in controlling the cellular progesterone concentration, which is an essential hormone impeccable for maintenance of pregnancy (Ji et al., 2004; Lewis et al., 2004; Piekorz et al., 2005). These studies indicate that inhibition of progesterone metabolism mediated by AKR1C1 is a promising strategy for preventing pre-term birth. We therefore propose AKR1C1 as a new potential therapeutic target in pregnancy maintenance.

To date, a variety of non-competitive and competitive inhibitors of AKR1C1 have been identified. The representative non-competitive inhibitors of AKR1C1 are benzodiazepines. For instance, medazepam potently inhibited AKR1C1. A series of phthalimide, pyrimidine and anthranilic acid derivatives were further synthesized by Brozic et al. (2009). Meanwhile, compounds that have a core structure of steroid carboxylate and flavones are AKR1C1 competitive inhibitors. 3-bromo-5-phenylsalicylic acid (BPSA) is a lead inhibitor for AKR1C1. It potently inhibited the progesterone metabolism of bovine aortic endothelial cells overexpressing AKR1C1 (IC_50_ = 460 nM) (El-Kabbani et al., 2009). Given the fact that BPSA selectively inhibited AKR1C1 activity, it seems a promising strategy to apply BPSA as a progesterone metabolism inhibitor. However, BPSA is an artificially synthetic compound, resulting in expensive cost and unclarified risks. Thus identifying new inhibitors selective for AKR1C1 from natural compound with minor side effects which can be used extensively and safely represents an attractive opportunity for prevention of pre-term birth induced with low progesterone level.

In the current study, we screened a natural compound library in an effort to identify novel AKR1C1 inhibitors. The screening by enzyme activity assay found liquiritin as a newly discovered AKR1C1 inhibitor that is as potent as BPSA. Furthermore, we demonstrated that liquiritin efficiently inhibited progesterone metabolism mediated by AKR1C1 *in vivo*.

## Materials and Methods

### Enzyme Purification

GST-tagged recombinant AKR1C isoforms were cloned into pGex-4T-1 vectors and expressed in *Escherichia coli* JM109 and purified as described (Matsuura et al., 1998a,b; Shiraishi et al., 1998). GST-tagged recombinant AKR1C isoforms expressed JM109 was grown in Luria-Bertani broth (100 μg⋅ml-1 ampicillin, pH = 7.0) at 37°C with orbital shaking at 220 rpm. When cells achieved an OD_600__nm_ between 0.4 and 0.6, 0.3 mM final concentration of isopropyl β-D-1-thiogalactopyranoside (IPTG) was added to the cultures and incubated for an additional 12 h at 25°C. Cells were harvested by centrifugation at 8000 *g* for 10 min, and the cell pellets were stored at -80°C for storage. The pellets were resuspended in 1 × PBS, pH = 7.4 and lysed by ultrasonication at ice-cold temperature using an UH-03 union instrument. The purification of glutathione-S-transferase (GST)-fusion AKR1C1, AKR1C2, and AKR1C3 were then carried out using ÄKTA systems from GE Healthcare as described in Handbook. The recombinant proteins were eluted in 50 mM Tris–HCl (pH = 8.0) with 20 mM reduced glutathione, fresh preparedly. Protein concentrations were determined using the Bradford protein assay kit. All proteins were aliquoted and stored at -80°C containing 20% glycerol.

### Assay of Enzyme Activity

The kinetic constants of AKR1C isoforms was assayed by detecting the rate of change in NADPH fluorescence (at 340 nM). The assays were carried out in a 200 μl volume containing 20 μg recombinant proteins, varying concentration of s-tetralol, 0.2 mM NADP and 100 mM potassium phosphate buffer, pH = 7.4, at 37°C, as described by El-Kabbani (Dhagat et al., 2008). To screen for the indicated inhibitor, the assay mixture was added with additional DMSO or 2 μM compounds. The IC_50_ values for the inhibitors were detected with s-tetralol (23.47 μM for AKR1C1, 103.30 μM for AKR1C2, and 851.10 μM for AKR1C3) and various concentrations of inhibitors. The inhibition patterns were determined by fitting initial velocity and inhibitor concentration into inhibition models using Graphpad Prism 6.0. All inhibition data were obtained from single experiments, with samples conducted in duplicate.

### Molecular Docking

The docking studies of liquiritin and BPSA with AKR1C1, AKR1C2, AKR1C3, and AKR1C4 were performed using the AutoDock Vina program (Version 1.1.2) (Trott and Olson, 2010). Protein Data Bank (PDB) entries 1MRQ (Couture et al., 2003), 1IHI (Jin et al., 2001), 3R43 (Flanagan et al., 2012), and 2FVL (Waxman et al., 1988) were selected as the AKR1C1, AKR1C2, AKR1C3, and AKR1C4, respectively. Auto Dock Tools (Sanner et al., 2007) were applied for preparation of receptors and ligands. For preparing receptors, the solvent molecules and original ligands from the crystals were removed, with retaining one molecule of the respective enzyme and the NADP+ cofactor. And the binding pocket was defined to cover around the original ligand within 15 Å radius sphere. For preparing ligands, the 3D-structure of ligands were generated by ChemBio3D and optimized under a Minimization field using the MM2 forcefield initially, and then processed by Auto Dock Tools via adding hydrogens, and assigning Gasteiger charges. The docking experiments were carried out taking into account the flexibility of ligands only. Each run produced nine docking poses which were ranked according to scoring energy. Those conformations with the lowest energy were selected and visualized by PyMOL.

### Immunoblotting Analysis

Immunoblotting analysis of proteins in cell lysates was performed as previously described (Zhou et al., 2016). Primary antibodies were as follows: GAPDH (Santa Cruz, sc-25778), AKR1C1 (GeneTex, GTX105620), and GST (Santa Cruz, sc-138).

### Evaluation of Inhibitors in the Cells

HEC-1-B cell line was purchased from the Cell Bank of the Chinese Academy of Sciences (Shanghai, China). HEC-1-B cell was cultured in DMEM (Gibco, Grand Island, New York, United States) supplemented with 10% fetal bovine serum at 37°C in a 5% CO2 incubator. In all experiments, the cells were used at passages 4 to 10. The cells were transfected with AKR1C1 plasmid using jet PRIME reagent (Polyplus) according to manufacturer’s instructions. The transfected cells were pretreated for 6 h with liquiritin or BPSA in serum-free growth medium prior to incubating for 6 h with 5 μM progesterone. The culture media were collected immediately and 6 h later. The metabolite of progesterone were quantified on a LC-MS using a Agilent HC C18 column (150 mm × 4.6 mm, 5 μm particle size) as described by Ishikura et al. (2005). A LC-20A High Performance liquid chromatography (Shimadzu, Japan) with UV detector was used for analyzing progesterone cultured from the cells. Ultra pure water from PureLab Option (ELGA, United Kingdom) was used as mobile phase A. The mobile phase B consisted of methanol and acetonitrile at a ratio of 1:7. The mobile phase used was a mixture of mobile phase A and mobile phase B in an isocratic program as 3:7 running on an Agilent HC C18 column (150 mm × 4.6 mm, 5 μm particle size) at a constant temperature of 35°C. The flow rate was 1.0 mL/min and the detection wavelength was 245 nm. The whole analytical period was 10 min for each sample. The standard (progesterone) was dissolved in methanol. Calibration standards were prepared by adding 5 μL of each spiking solution to 45 μL of drug-free cell culture fluid in order to obtain the following concentrations: 0.2, 0.5, 1.0, 2.0, and 5.0 μg/mL. The corresponding calibration curve was *y* = 29866.3x – 654.964. The QC samples were prepared by the same method mentioned above to reach the final concentrations: 0.6, 2.0, and 4.0 μg/mL. All samples were treated by methanol to precipitate the impurity. The mixture was then vortexed at 1600 rpm for 10 min, and centrifuged at 12000 rpm for 10 min. The upper layer was collected for analysis.

### Statistical Analysis

All results were expressed as mean ± s.d. For quantitative analysis, experiments were repeated three times and the differences among means were significant determined by Student t-text (^∗^*p* < 0.05; ^∗∗^*p* < 0.01; ^∗∗∗^*p* < 0.001).

## Results

### Liquiritin Is a Novel AKR1C1 Inhibitor

AKR1C1 played crucial roles in progesterone metabolism, and contributed to the treatment of pregnancy maintenance (Sinreih et al., 2015). We designed the study of finding AKR1C1 inhibitor based on purification of AKR1C1 enzymes from *E. coli* followed by conducting enzymatic assay, which is a classic method in screening inhibitors of AKR1C1 (El-Kabbani et al., 2010; Zheng et al., 2018). Therefore, we purify the AKR1C proteins coding genes of human sapiens for our study, which should almost resemble to endogenous human AKR1C proteins. Besides, several studies successfully reveal the AKR1C1 crystal structure using AKR1C1 recombinant proteins together with inhibitors screened by enzymatic studies, which suggests that recombinant AKR1C1 could at least reflect enzyme activity of endogenous AKR1C1 (Dhagat et al., 2008; Zhao et al., 2015).

In order to identify compounds effectively inhibiting progesterone metabolism mediated by AKR1C1, we established a library of 67 natural compounds, and screened for AKR1C1 inhibitor. Firstly, we overexpressed recombinant human AKR1C in *E. coli* in the form of a GST-fusion protein, and then purified it by affinity chromatography on glutathione-Sepharose. Secondly, we examined the purity of AKR1C by SDS-PAGE, which revealed a band with an approximate molecular weight of 64 kDa (Supplementary Figure S1A). The homogenous recombinant AKR1C1 catalyzed s-tetralol with an apparent *K_M_* of 23.47 μM. And the catalytic efficiency is 0.53 min^-1^/μM^-1^, which is comparable to that have been reported by others (Table 1), suggesting the proper enzyme activity of AKR1C1 (Brozic et al., 2012).

**Table 1:** *K*_M_ and *k*_cat_ value of recombinant AKR1C isoform proteins using s-tetralol as substrate by NADPH assay.

Kinetic constants for the conversion of the S-tetralol by AKR1C1-AKR1C3				
	**S-tetralol/NADP+**			
	
**Coenzyme/Substrate**	***K*_M_ (mM)**	***V*_max_ (nmol/min/mg)**	***K*_cat_ (min^-1^)**	***K*_cat_/*K*_M_ (min^-1^mM^-1^)**

GST-AKR1C1	23.47	203.10	12.39	0.53
GST-AKR1C2	103.30	94.58	5.77	0.06
GST-AKR1C3	851.10	543.90	33.18	0.04


To screen for the inhibitors of AKR1C1, we developed a readily accessible indirect NADPH generating-based assay that can be used for high-throughput inhibitors screening. The assay utilizes the pan-AKR1C substrate s-tetralol and NADP^+^ as cofactors and measures AKR1C activity indirectly by determining levels of NADPH. As one of the most potent AKR1C1 inhibitor, BPSA was included as a system positive control. Wells were included containing enzyme and no inhibitor or 2 μM inhibitor. The screen identified liquiritin as a potential AKR1C1 inhibitor (Supplementary Table S1).

**FIGURE 1:**
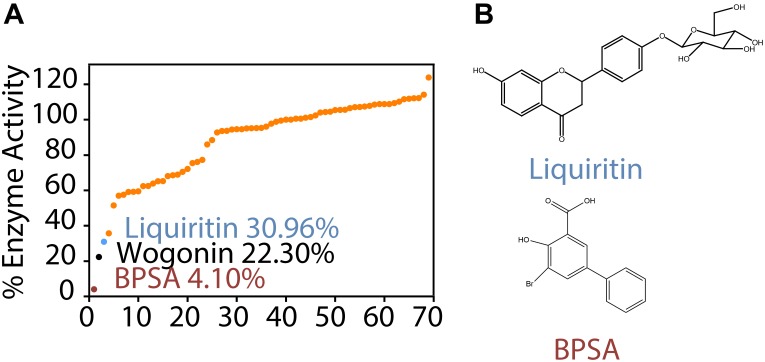
Identification of liquiritin as a novel AKR1C1 inhibitor. **(A)** Enzyme activity assay showed that liquiritin exerted inhibitory effect for recombinant AKR1C1 protein at 2 μM. **(B)** Structure of liquiritin and BPSA.

As shown in Figure 1A, BPSA efficiently decreased the enzyme activity of AKR1C1 down to 4.10%, implying the reliability of our enzyme activity system. On this premise, we observed that both wogonin (22.30% inhibition) and liquiritin (33.96% inhibition) exhibited remarkable inhibitory effect over AKR1C1 enzyme activity. As wogonin had already been reported to inhibit elevated AKR1C1 expression induced by IL-6 (Wang et al., 2007), we were thus inspired to investigate the inhibition effect of liquiritin over AKR1C1 enzyme activity (Figure 1B). Each experiment was repeated three times and the differences among means were significant determined by Student t-text (^∗^*p* < 0.05; ^∗∗^*p* < 0.01; ^∗∗∗^*p* < 0.001).

### Liquiritin Selectively Inhibits AKR1C1

**FIGURE 2:**
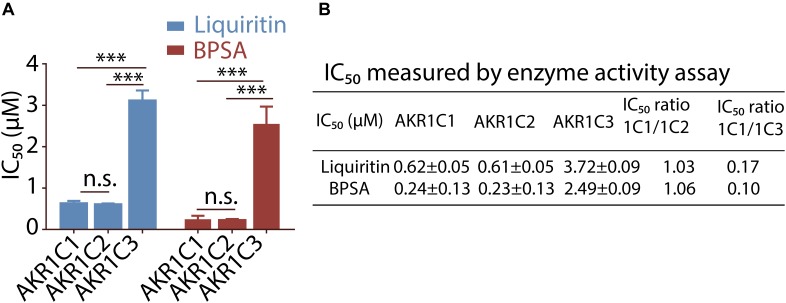
Selectivity of liquiritin and BPSA evaluated by enzyme activity assay. **(A)** The isoform specificity of liquiritin and BPSA against recombinant AKR1C proteins using the NADPH assay. **(B)** The IC_50_ of liquiritin and BPSA for recombinant AKR1C isoforms was determined using the NADPH assay (see section “Materials and Methods”). ^∗^*p* < 0.05; ^∗∗^*p* < 0.01; ^∗∗∗^*p* < 0.001.

To investigate selectivity of liquiritin for AKR1C, we purified recombinant AKR1C2 and AKR1C3 and tested the kinetic parameters for s-tetralol reduction (Table 1 and Supplementary Figure S1B). We then evaluated the ability of liquiritin to inhibit AKR1C enzyme activity using an *in vitro* enzyme assay monitoring generating of NADPH as a readout (Supplementary Figure S2), where it displayed an IC_50_ of 0.62 ± 0.05 μM and 0.61 ± 0.05 μM for AKR1C1 and AKR1C2, respectively when using s-tetralol as a substrate (Figure 2A,B). It was evident from the results that liquiritin was as potent and as selective comparing to BPSA for the ability to inhibit AKR1C1 and AKR1C2 enzyme activity. Each experiment was repeated three times and the differences among means were significant determined by Student t-text (^∗^*p* < 0.05; ^∗∗^*p* < 0.01; ^∗∗∗^*p* < 0.001).

### Liquiritin Is a Competitive AKR1C1 Inhibitor

**FIGURE 3:**
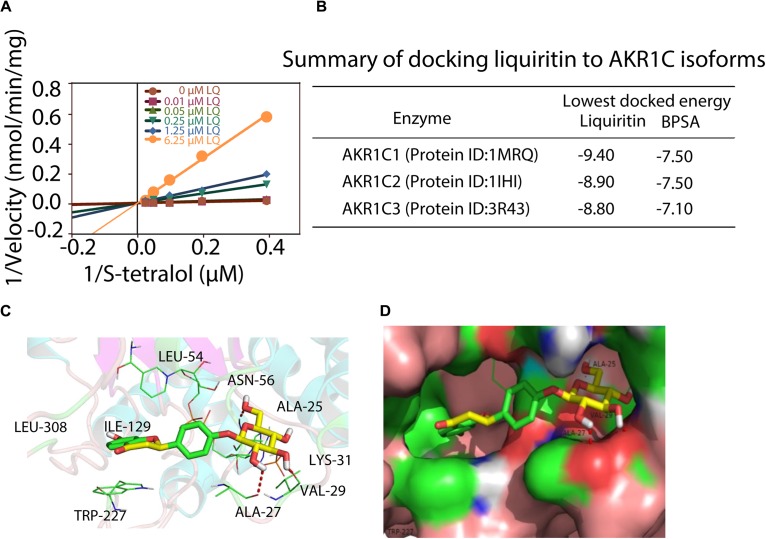
Characterization of liquiritin. **(A)** Kinetic study of liquiritin against AKR1C1 with s-tetralol concentration variation. **(B)** Summary of docking liquiritin to AKR1C isoforms. **(C,D)** Docking of liquiritin into the active site of AKR1C1. Stereo view of the phytoestrogen binding site of AKR1C1, showing NADPH, liquiritin (stick) and the amino-acid residues of the active site. Three H-bonds were marked with red dashlines, and one π-π interaction was marked with blue dashline.

We next turned to investigate the mechanism for liquiritin mediated inhibition of AKR1C1 enzyme activity. Enzymatic studies demonstrated that liquiritin was a s-tetralol competitive inhibitor against AKR1C1 (Figure 3A). Liquiritin has a flavone molecular structure, closely resemble to the primary of AKR1C1 competitive inhibitors.

Therefore, this result is consistent with our knowledge about AKR1C1 competitive inhibitor. Furthermore, we performed molecular docking simulations of liquiritin and BPSA into AKR1C isoforms (Supplementary Figure S3). The results showed that liquiritin can enter the progesterone-binding region of the active site. In the position with the lowest docking energy (Figure 3B), the flavone part (i.e., 7-hydroxychroman-4-one) are located in the cave surrounded by Leu308, ILE-129, Trp227, Try24, Leu-54, and NADPH, and the phenyl ring engages a π-π stacking interaction with residue Trp-227 (Figure 3C). While the glucose moiety anchors toward the hole composed of residues C. This type of binding supports the importance of this glucose moiety, since this group can form three H-bonds with Ala-27, Val-29, and Ala-25, respectively (Figure 3D). The flavone part resembles the hypothetical catalytic orientation of progesterone in the AKR1C1 active site.

Given the complexity of cellular activity, there still might be a significant difference when applied liquiritin to cells to test its function on inhibiting progesterone metabolism. So the next important thing is to evaluate the progesterone metabolism inhibition ability of liquiritin via cellular experiments. Each experiment was repeated three times and the differences among means were significant determined by Student t-text (^∗^*p* < 0.05; ^∗∗^*p* < 0.01; ^∗∗∗^*p* < 0.001).

### Liquiritin Inhibits Progesterone Metabolism via Inhibiting AKR1C1

**FIGURE 4:**
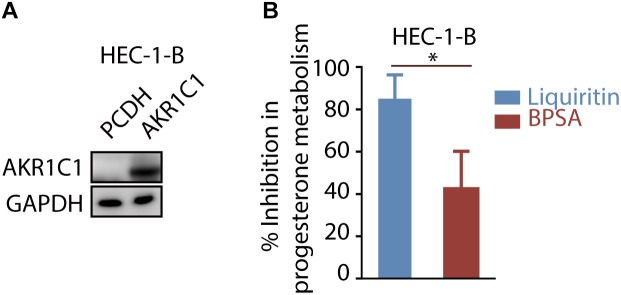
Liquiritin inhibits progesterone metabolism via AKR1C1. **(A)** HEC-1-B cells were transiently transfected with PCDH or AKR1C1. Cell lysates were used for western blotting with indicated antibodies. **(B)** The HEC-1-B cells were cultured for 6 h in the medium containing 5 μM progesterone in the presence of 50 μM liquiritin or BPSA. ^∗^*p* < 0.05; ^∗∗^*p* < 0.01; ^∗∗∗^*p* < 0.001.

In order to further evaluate the ability of liquiritin to inhibit progesterone metabolism mediated by AKR1C1 at cellular level, HEC-1-B cells were transfected with AKR1C1 plasmid. We intend to evaluate the progesterone metabolism inhibitory effect of liquiritin at cellular level, which is more relevant to *in vivo* situation instead of enzymatic study, considering its clinical application in maintaining progesterone level. Therefore, we choose to transfect cells with AKR1C1 gene for cellular study. The expression of AKR1C1 in the cells was identified by western blot assay (Figure 4A). Liquiritin effectively inhibited progesterone metabolism via inhibiting AKR1C1. As shown in Figure 4B, 50 μM liquiritin led to significant inhibition of progesterone metabolism for nearly 85.00%. In contrast, at the same condition, BPSA only displayed 42.50% inhibition of progesterone metabolism. Each experiment was repeated three times and the differences among means were significant determined by Student *t*-text (^∗^*p* < 0.05; ^∗∗^*p* < 0.01; ^∗∗∗^*p* < 0.001).

## Discussion

In the present study, we report herein the identification of liquiritin as a novel inhibitor of AKR1C1. Molecular docking experiments in combination with enzyme activity assays suggest that liquiritin might exert its inhibitory activity by targeting the Ala-27, Val-29, Lys-31, Ala-25, and Asn-56 residues of AKR1C1. Cellular tests reveal that liquiritin treatment significantly reduces the progesterone metabolism mediated by AKR1C1, suggesting that liquiritin is capable of interfering with progesterone level in cells. Our study thus has identified the liquiritin as a novel natural compound for inhibiting AKR1C1 enzyme activity and its potential function in preventing pre-term birth induced with lower progesterone.

Licorice root, has already been widely used in China as a herbal medicine for preventing miscarriage while the mechanism remains to be elucidated (Fu et al., 2005). Our current study has for the first time revealed that as the major component of licorice root, liquiritin could inhibit progesterone metabolism by inhibiting AKR1C1 activity and therefore prevent pre-term birth induced by low progesterone level. Liquiritin has a variety of pharmacology activities, including antioxidant activities and anti-inflammatory activities (Sun et al., 2010; Yu et al., 2015; Nakatani et al., 2017). Our above-mentioned data demonstrated that liquiritin effectively inhibited the metabolism of progesterone in HEC-1-B cells. Therefore, it is reasonable to suppose that liquiritin could exclusively function on HEC-1-B cells by inhibiting AKR1C1 enzymatic activity. Besides, liquiritin is a component of licorice root, which is a kind of resourceful plant. Therefore, comparing to chemical inhibitors of AKR1C1, liquiritin possesses great advantage when put into application due to its broad source.

In addition to AKR1C1 enzymatic activity inhibition, liquiritin was also implicated in inhibiting AKR1C2 and AKR1C3 activity. As showed in other studies, both elevated AKR1C2 and AKR1C3 could lead to enhanced progesterone metabolism (Ji et al., 2004; Hevir et al., 2011). In this case, the broad inhibition effect of liquiritin on AKR1C families may be an advantage when applied as a progesterone metabolism inhibitor.

Taken together, we have identified liquiritin as a potent AKR1C1 inhibitor extracted from natural plant licorice. Moreover, we have shown that liquiritin is efficacious in inhibiting the metabolism of progesterone at cellular levels. Thus our current study provide the first evidence that liquiritin could be applied to prevent pre-term birth caused by low progesterone levels.

## Data Availability

The raw data supporting the conclusions of this manuscript will be made available by the authors, without undue reservation, to any qualified researcher.

## Author Contributions

BY, HZ, and QH conceived and designed the studies. CZ and DZ performed most of the experiments, the statistical analysis, and contributed to the writing of the manuscript. JY and XD provided technical support.

## Conflict of Interest Statement

The authors declare that the research was conducted in the absence of any commercial or financial relationships that could be construed as a potential conflict of interest.
