# Supplementary material for: Liquiritin, as a Natural Inhibitor of AKR1C1, Could Interfere With the Progesterone Metabolism
Source: Front Physiol. 2019 Jul 3;10:833. doi: 10.3389/fphys.2019.00833 (PMC6616128; doi:10.3389/fphys.2019.00833)
Supplement: Supplementary file 1 [file Image_1.pdf]

Supplementary Figure 1

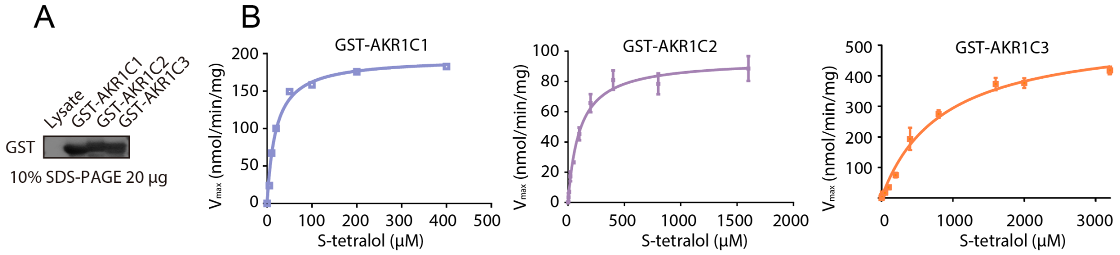

Supplementary Figure 1. Purification and kinetic constants of recombinant AKR1C isoforms. (A) SDS-PAGE results of recombinant AKR1C isoforms. **(B)** K_M and k_{cat} of recombinant AKR1C isoforms.

Supplementary Figure 2

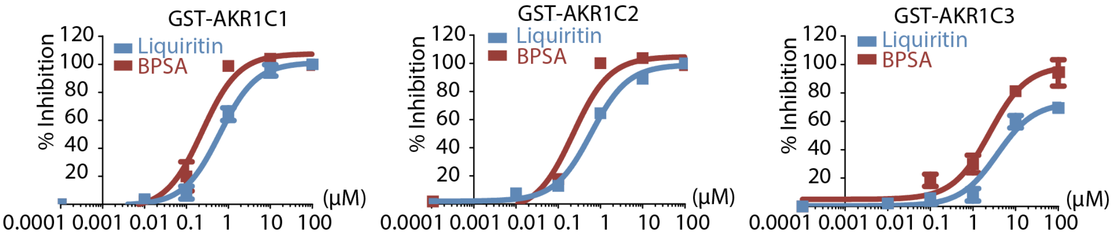

Supplementary Figure 2. Dose-dependent inhibition of AKR1C isoforms by liquiritin and BPSA.

Supplementary Figure 3

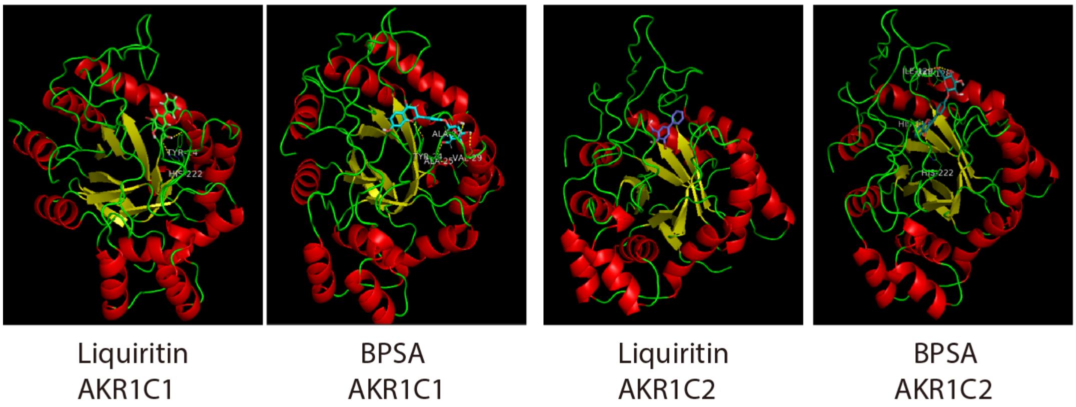

Supplementary Figure 3. Docking of liquiritin or BPSA into the active site of AKR1C isoform proteins.

Supplementary Table 1

	Compound	slope	nmol/min/mg	ratio	% inhibition
	CON	0.0122	192.295568	1	0
	BPSA	0.0005	7.881	0.041	95.9
1	Wogonin	0.00272	42.8725	0.223	77.7
2	Liquiritin	0.003777	59.5351	0.3096	69.04
3	Glycitin	0.00436	68.722	0.3574	64.26
4	Curcumin	0.006283	99.03	0.515	48.5
5	Picroside Ⅱ	0.006957	109.658	0.5703	42.97
6	(−)-Epicatechin gallate	0.00702	110.6488	0.5754	42.46
7	Tenuifolin	0.007214	113.7111	0.5913	40.87
8	Leonurine hydrochloride	0.007226	113.8912	0.5923	40.77
9	Costundide	0.007263	114.4767	0.5953	40.47
10	Astragalin	0.007614	120.0159	0.6241	37.59
11	Bilobalide	0.007623	120.151	0.6248	37.52
12	Isoquercitrin	0.007797	122.898	0.6391	36.09
13	Daidzein	0.007957	125.4199	0.6522	34.78
14	Ginkgolide B	0.007969	125.6001	0.6532	34.68
15	Ginkgolide C	0.008309	130.9591	0.681	31.9
16	Salvianolic acid B	0.008371	131.9499	0.6862	31.38
17	Ginkgolide A	0.008406	132.4903	0.689	31.1
18	Glycitein	0.0086	135.5526	0.7049	29.51
19	Hydroxysafflor yellow A	0.008806	138.7951	0.7218	27.82
20	Bavachinin A	0.009203	145.0548	0.7543	24.57
21	3,5,7-Trihydroxyflavone	0.00928	146.2707	0.7607	23.93
22	Mangiferin	0.009429	148.6125	0.7728	22.72
23	Sodium Danshensu	0.0105	165.3652	0.86	14
24	Isoimperatorin	0.0108	170.1838	0.885	11.5
25	3-Hydroxy-4-methoxycinnamic acid	0.0113	178.38	0.9276	7.24
26	Quercitrin	0.0114	180.0013	0.9361	6.39
27	Bisdemethoxycurcumin	0.0114	180.2715	0.9375	6.25
28	Saikosaponin A	0.0115	181.6675	0.9447	5.53
29	Saikosaponin D	0.0115	181.8927	0.9459	5.41
30	Kaempferide	0.0115	181.9828	0.9464	5.36
31	Psoralen	0.0116	182.8834	0.9511	4.89
32	Calycosin	0.0116	183.0185	0.9518	4.82
33	Pseuginsenoside F11	0.0116	183.1536	0.9525	4.75
34	Peimine	0.0116	183.3338	0.9534	4.66
35	Imperatorin	0.0117	184.7298	0.9607	3.93
36	Psoralidin	0.0119	187.9723	0.9775	2.25
37	5-Methoxypsoralen	0.0121	190.1339	0.9888	1.12

38	(−)-Epigallocatechin gallate	0.0121	191.3949	0.9953	0.47
39	Oxypeucedanin	0.0122	192.3856	1.0005	-0.05
40	Ginsenoside CK	0.0123	193.3764	1.0056	-0.56
41	Hyperoside	0.0123	193.4665	1.0061	-0.61
42	Acteoside	0.0123	194.5022	1.0115	-1.15
43	Prim-O-glucosylcimifugin	0.0124	195.2678	1.0155	-1.55
44	Isorhamnetin	0.0125	197.0241	1.0246	-2.46
45	Nobiletin	0.0127	200.1315	1.0407	-4.07
46	Jatrorrhizine Hydrochloride	0.0127	200.7169	1.0438	-4.38
47	Emodin-3-methyl ether	0.0128	201.0772	1.0457	-4.57
48	Albiflorin	0.0129	202.8786	1.055	-5.5
49	Arctigenin	0.0129	203.0137	1.0557	-5.57
50	Genistin	0.0129	203.1488	1.0564	-5.64
51	Daidzin	0.013	204.9952	1.066	-6.6
52	Echinacoside	0.0131	205.9859	1.0712	-7.12
53	(−)-Epigallocatechin	0.0131	206.076	1.0717	-7.17
54	Epigallocatechin	0.0131	206.6164	1.0745	-7.45
55	(+)-Catechin hydrate	0.0132	207.427	1.0787	-7.87
56	Schisantherin A	0.0132	208.778	1.0857	-8.57
57	Schisandrin A	0.0133	209.2284	1.0881	-8.81
58	Tangeretin	0.0133	209.3185	1.0885	-8.85
59	Wogonoside	0.0133	209.3635	1.0888	-8.88
60	Shikonin	0.0134	210.4443	1.0944	-9.44
61	Dioscin	0.0134	211.8854	1.1019	-10.19
62	Epicatechin	0.0136	214.5874	1.1159	-11.59
63	Glabridin	0.0136	215.1279	1.1187	-11.87
64	Schisandrol B	0.0137	215.6683	1.1215	-12.15
65	Schisandrol A	0.0137	215.8934	1.1227	-12.27
66	Fangchinoline	0.0139	219.4962	1.1415	-14.15
67	Schizandrin B	0.0151	238.2754	1.2391	-23.91

Supplementary Table 1. 67 natural compounds were screened for recombinant AKR1C1 protein inhibitory activity effect at 2 µM.
